# Elbasvir Inhibits Hepatitis E Virus Internalization and, in Combination with Ribavirin, Achieves Sustained Viral Suppression In Vitro

**DOI:** 10.3390/pathogens15060607

**Published:** 2026-06-05

**Authors:** Putu Prathiwi Primadharsini, Shigeo Nagashima, Masaharu Takahashi, Kazumoto Murata, Hiroaki Okamoto

**Affiliations:** Division of Virology, Department of Infection and Immunity, School of Medicine, Jichi Medical University, Shimotsuke 329-0498, Tochigi, Japan; thiwik8@jichi.ac.jp (P.P.P.); shigeon@jichi.ac.jp (S.N.); mtaka84@jichi.ac.jp (M.T.); kmurata@jichi.ac.jp (K.M.)

**Keywords:** hepatitis E virus, drug screening, elbasvir, ribavirin, cell culture

## Abstract

Hepatitis E virus (HEV) infection is generally self-limiting in immunocompetent individuals but may progress to chronic infection in immunocompromised patients, underscoring the need for effective antiviral therapies. Although ribavirin is currently used off-label for HEV treatment, its associated adverse effects highlight the need for safer alternatives. In this study, we screened an anti-viral compound library comprising 800 compounds using three HEV reporter systems designed to target distinct stages of the viral life cycle. Candidate compounds were further evaluated in PLC/PRF/5 cells using both acute and chronic infection models with wild-type genotype 3 HEV (HEV-3). Antiviral activity was assessed by measuring HEV RNA levels in culture supernatants. Elbasvir, a known inhibitor of hepatitis C virus (HCV) non-structural protein 5A (NS5A), was identified as the most potent candidate. Although multiple compounds showed inhibitory effects in reporter assays, only elbasvir achieved sustained suppression of HEV growth in long-term culture, reducing HEV RNA levels to below the limit of detection. In a chronic infection co-culture model, elbasvir maintained antiviral activity at non-cytotoxic concentrations. Time-of-addition analysis demonstrated that elbasvir inhibits an early step in the viral life cycle, specifically viral internalization. Furthermore, combination with ribavirin enhanced antiviral efficacy, resulting in sustained viral suppression without detectable cytotoxicity and exhibiting an additive interaction. Collectively, these findings identify elbasvir as a promising candidate for repurposing as an anti-HEV drug and support a combination strategy targeting distinct steps of the viral life cycle.

## 1. Introduction

Hepatitis E virus (HEV), a member of the family *Hepeviridae*, subfamily *Orthohepevirinae*, genus *Paslahepevirus*, species *Paslahepevirus balayani*, is a positive-sense single-stranded RNA virus [1]. In infected hosts, HEV circulates in the bloodstream predominantly as membrane-associated quasi-enveloped particles (eHEV), whereas non-enveloped virions (neHEV) are shed in feces [2]. The HEV genome is approximately 7.2 kilobases (kb) in length and contains three open reading frames (ORF1–3), flanked by short 5’- and 3’-untranslated regions (UTRs) and terminated by a poly(A) tail [3,4]. ORF1 encodes a non-structural polyprotein involved in viral replication and contains several functional domains, including the methyltransferase and Y domain (MetY), fatty acid- binding domain-like domain (FABD-like), hypervariable region (HVR), X (macro) domain, helicase domain (Hel), and RNA-dependent RNA polymerase domain (RdRp) [5,6,7,8,9,10]. ORF2 encodes the viral capsid protein, which exists in infectious, glycosylated, and cleaved forms and plays essential roles in virion assembly and host cell attachment. In addition, ORF2 serves as the primary target of neutralizing antibodies [11,12,13,14]. ORF3 encodes a multifunctional phosphoprotein that facilitates virion egress and exhibits viroporin activity through ion channel formation [15,16]. Among the eight recognized HEV genotypes, HEV genotypes 1–4 (HEV-1 to HEV-4) are the most prevalent worldwide [17]. HEV-1 and HEV-2 are restricted to humans, whereas HEV-3 and HEV-4 are zoonotic and circulate primarily in pigs and wild boars [18,19].

HEV infection is a major cause of acute viral hepatitis worldwide, with an estimated 19.47 million infections and 3450 deaths reported in 2021 [20]. Although HEV infection is generally self-limiting in immunocompetent individuals, severe disease can occur in vulnerable populations, including acute liver failure in pregnant women [21] and chronic infection in immunocompromised patients [22,23]. Chronic HEV infection remains a significant clinical challenge, particularly among solid-organ transplant recipients and other immunocompromised individuals. Ribavirin is currently used off-label as the primary treatment for chronic HEV infection and has demonstrated clinical efficacy [24,25]. However, its use is frequently limited by adverse effects, most notably hemolytic anemia [26,27,28], underscoring the need for alternative therapies options.

**Figure 1:**
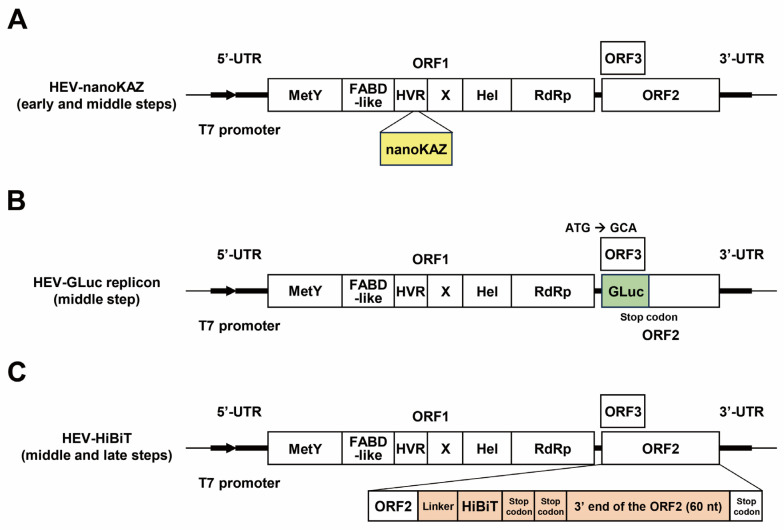
Schematic representation of three distinct HEV reporter systems used for anti-viral compound library screening. (**A**) HEV-nanoKAZ covering the early and middle steps of the HEV life cycle [30]; (**B**) HEV-GLuc replicon, representing the middle step [31]; and (**C**) HEV-HiBiT, covering the middle and late steps [32]. MetY, the methyltransferase and Y domain; FABD-like, the fatty-acid binding domain-like domain; HVR, the hypervariable region; X, the X or macro domain; Hel, the helicase domain, and RdRp, the RNA-dependent RNA polymerase domain.

Targeting multiple stages of the viral life cycle represents a promising strategy for antiviral development [29]. The HEV life cycle can be broadly divided into early (attachment and internalization), middle (translation and RNA replication), and late (virus particle formation and virion release) stages. To facilitate stage-specific antiviral evaluation, we recently established three HEV reporter systems: HEV-nanoKAZ [30], HEV-GLuc replicon [31], and HEV-HiBiT [32], which monitor the early, middle, and late stages of the HEV life cycle, respectively (Figure 1). Together, these platforms enable systematic assessment of compounds targeting distinct stages of HEV infection.

In the present study, we performed a comprehensive screening of an antiviral compound library comprising 800 compounds using these three HEV reporter systems. Candidate compounds were subsequently evaluated in PLC/PRF/5 cells using both acute and chronic infection models with wild-type HEV-3. Through this screening approach, we identified elbasvir, a hepatitis C virus (HCV) non-structural protein 5A (NS5A) inhibitor, as a potent inhibitor of HEV infection. Elbasvir exhibited sustained antiviral activity during long-term culture and inhibited viral internalization. Furthermore, combination treatment with ribavirin enhanced antiviral efficacy, supporting a dual-target therapeutic strategy for the treatment of HEV infection.

## 2. Materials and Methods

### 2.1. Cell Culture

PLC/PRF/5 cells (ATCC No. CRL-8024; American Type Culture Collection, Manassas, VA, USA) were maintained in Dulbecco’s modified Eagle’s medium (DMEM; Thermo Fisher Scientific, Waltham, MA, USA) supplemented with 10% heat-inactivated fetal bovine serum (FBS; Thermo Fisher Scientific Inc.) at 37 °C in a humidified atmosphere containing 5% CO_2_, as previously described [33]. For maintenance of HEV-infected cells, the culture medium was additionally supplemented with 1% dimethyl sulfoxide (DMSO; Fujifilm Wako, Osaka, Japan).

### 2.2. Viruses

Culture supernatants containing the cell culture-adapted HEV-3 JE03-1760F strain (passage 26; 1.5 × 10^8^ copies/mL) were used as the virus inoculum [34].

### 2.3. Compounds and Reagents

An antiviral compound library comprising 800 compounds was purchased from TargetMol (Boston, MA, USA). Because the remaining volumes of several compounds in the library stock were insufficient for subsequent analyses, the hit compounds elbasvir, cenicriviroc, samatasvir, hydroxychloroquine sulfate, azvudine, bemnifosbuvir hemisulfate, and RO8191 were additionally purchased from the same supplier. Ribavirin and sucrose were obtained from Fujifilm Wako.

### 2.4. Compound Library Screening and Validation of Anti-HEV Activity

**Figure 2:**
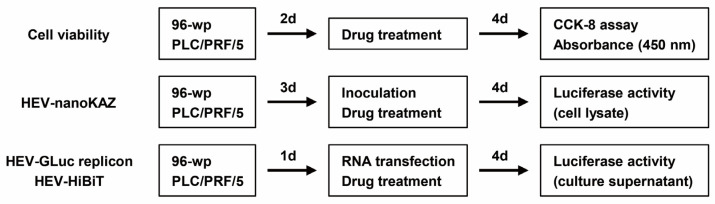
Experiment workflow for the cell viability assay and the HEV-nanoKAZ, HEV-GLuc replicon, and HEV-HiBiT assays.

Primary screening of the antiviral compound library and subsequent validation of hit compounds were performed using three HEV reporter systems: HEV-nanoKAZ [30], the HEV-GLuc replicon [31], and HEV-HiBiT [32], as previously described (Figure 1 and Figure 2). For primary screening, all compounds were tested at a final concentration of 10 μM.

Compounds meeting the predefined selection criteria were subjected to secondary screening at 1 μM. Candidate hit compounds identified from the secondary screening were further evaluated for antiviral activity over a range of concentrations using the respective reporter systems.

Luciferase activity was measured using a TriStar2 LB942 multimode plate reader (Berthold Technologies, Bad Wildbad, Germany). Luminescence values were expressed as relative light units (RLUs) and normalized to those of the DMSO-treated cells, which served as the vehicle control.

### 2.5. Cell Viability Assay

Cell viability was assessed using the Cell Counting Kit-8 (Dojindo Laboratories, Kumamoto, Japan) according to the manufacturer’s instructions. For the pre-culture viability assay, PLC/PRF/5 cells were seeded into 96-well plates (Thermo Fisher Scientific) and incubated at 37 °C for 48 h. The cells were then treated with the indicated concentrations of compounds prepared in 1% DMSO and incubated for an additional 96 h at 37 °C. Subsequently, the water-soluble tetrazolium salt (WST-8) reagent was added to each well, and the plates were incubated for 2 h at 37 °C. Absorbance at 450 nm was measured using an iMark microplate reader (Bio-Rad Laboratories, Hercules, CA, USA). Cell viability was normalized to that of DMSO-treated cells (vehicle control).

For the post-culture cell viability assay, cells harvested at the final experimental time point were reseeded into 96-well plates and incubated at 35.5 °C for 96 h. WST-8 reagent was then added, followed by incubation for 2 h at 37 °C, and absorbance at 450 nm was measured as described above.

A viability threshold of 80% was used as the cut-off criterion in all assays.

### 2.6. Evaluation of Antiviral Efficacy in an HEV Cell Culture System (Acute Infection Model)

PLC/PRF/5 cells monolayers cultured in 24-well plates (Thermo Fisher Scientific Inc.) were inoculated with 5 × 10^4^ copies of HEV-3 in FBS-free culture medium containing the indicated concentrations of compounds dissolved in 1% DMSO. After incubation at 37 °C for 2 h, the cells were washed five times with phosphate-buffered saline (PBS; pH 7.5) lacking Mg^2+^ and Ca^2+^ [PBS(−)]. Subsequently, 0.5 mL of culture medium containing the indicated concentrations of compounds in 1% DMSO was added to each well, and the cells were incubated at 35.5 °C.

Every other day, half of the culture medium was replaced with fresh medium containing the corresponding concentrations of compounds in 1% DMSO. At the indicated time points, culture supernatants were collected, centrifuged at 1300× *g* for 2 min at room temperature to remove cellular debris, and stored at −80 °C until further analysis.

### 2.7. Quantification of HEV RNA

Total RNA was extracted from culture supernatants using TRIzol-LS reagent (Thermo Fisher Scientific). HEV RNA levels were quantified by real-time reverse transcription (RT)-polymerase chain reaction (PCR) using a LightCycler system (Roche Diagnostics KK, Tokyo, Japan) and the LightCycler Multiplex RNA Virus Master (Roche Diagnostic KK), together with a primer-probe set targeting the overlapping region of ORF2 and ORF3, as previously described [35].

### 2.8. Evaluation of Antiviral Efficacy in an HEV Cell Culture System (Chronic Infection Model)

PLC/PRF/5 cells persistently infected with HEV-3 were maintained until HEV RNA levels in the culture supernatant reached a plateau (>10^8^ copies/mL). To establish the chronic infection model, naïve PLC/PRF/5 cells (1 × 10^5^ cells/well) were mixed with HEV-infected cells (1 × 10^2^ cells/well), seeded into 24-well plates, and incubated at 35.5 °C. At 48 h post-seeding, culture supernatants were removed and the cells were washed five times with PBS(−). Subsequently, 0.5 mL of culture medium containing the indicated concentrations of compounds dissolved in 1% DMSO was added to each well, and the cells were incubated at 35.5 °C. One-half of the culture medium was replaced every other day with fresh medium containing the corresponding concentrations of compounds.

At the indicated time points, culture supernatants were collected, centrifuged at 1300× *g* for 2 min at room temperature to remove cellular debris, and stored at −80 °C until analysis.

### 2.9. Assessment of Drug Synergy with Elbasvir and Ribavirin

The combined antiviral effects of elbasvir and ribavirin were evaluated by comparing the observed responses of drug combinations with the expected responses predicted under a no-interaction assumption. Drug synergy was assessed using the highest single agent (HSA) reference model implemented in SynergyFinder version 3.0 [36].

Under the HSA model, the expected effect of a drug combination is defined as the greater effect produced by either single agent at the corresponding concentrations. Luciferase activity was used as the primary endpoint to quantify antiviral activity. Synergy scores, dose–response curves, and interaction landscapes were generated using an 8 × 8 concentration matrix.

Elbasvir was tested at concentrations of 0, 0.1, 0.25, 0.5, 1, 2.5, 5, and 10 μM, based on the results of compound screening and subsequent antiviral evaluation. Ribavirin was tested at concentrations of 0, 1, 5, 10, 20, 40, 80, and 160 μM, as previously described [37]. Drug interactions were categorized according to the synergy score as antagonistic (<−10), additive (−10 to 10), or synergistic (>10).

### 2.10. Time-of-Addition Assay

PLC/PRF/5 cells were seeded in 96-well plates and incubated at 37 °C for 72 h to allow the formation of confluent monolayers.

For the viral inactivation assay, HEV-nanoKAZ (4 × 10^6^ copies/well) was preincubated with elbasvir (1 or 5 μM), sucrose (250 mM), or ribavirin (40 μM) in 1% DMSO for 1 h at 37 °C. The cells were washed twice with PBS(−), after which the virus–compound mixtures were inoculated onto the cells and incubated at 4 °C for 1 h to permit viral attachment, followed by incubation at 37 °C for 1 h to allow viral internalization. The cells were then washed once with PBS(−), and culture medium containing 1% DMSO was added before incubation at 35.5 °C.

For the viral attachment assay, the cells were washed twice with PBS(−) and inoculated with HEV-nanoKAZ in the presence of the indicated compounds in 1% DMSO. After incubation at 4 °C for 1 h, the cells were shifted to 37 °C for 1 h to allow viral internalization, washed once with PBS(−), and maintained in culture medium containing 1% DMSO at 35.5 °C.

For the viral internalization assay, the cells were washed twice with PBS(−) and inoculated with HEV-nanoKAZ at 4 °C for 1 h. Following removal of the inoculum, the indicated compounds dissolved in 1% DMSO were added, and the cells were incubated at 37 °C for 2 h. The cells were then washed once with PBS(−) and maintained in culture medium containing 1% DMSO at 35.5 °C.

In all assays, cell lysates were collected after 96 h of incubation at 35.5 °C, and intracellular luciferase activity was measured as described above. Cells treated with 1% DMSO alone served as vehicle controls.

### 2.11. Immunofluorescence Assay

PLC/PRF/5 cells subjected to viral inoculation and compound treatment were harvested at the final experimental time point, reseeded onto eight-well chamber slides (Watson, Tokyo, Japan), and incubated at 35.5 °C for 96 h. The cells were fixed in 4% (*v*/*v*) paraformaldehyde at room temperature for 15 min and permeabilized in 0.2% (*v*/*v*) Triton X-100 for 10 min at room temperature.

After washing with PBS(−), the cells were blocked with 1% bovine serum albumin (BSA) for 30 min. The cells were then incubated with a monoclonal antibody (MAb) against HEV ORF2 (H6225; 10 μg/mL in PBS[−] containing 1% BSA) [35] at 37 °C for 1 h. Following washing with PBS(−), the cells were incubated with an Alexa Fluor 488-conjugated anti-mouse IgG (Thermo Fisher Scientific; 2 μg/mL in PBS[−] containing 1% BSA) at 37 °C for 1 h.

Cell nuclei were counterstained with 4’,6-diamidino-2-phenylindole (DAPI; Thermo Fisher Scientific). The slides were mounted using Fluoromount-Plus mounting medium (Diagnostic Biosystems, Pleasanton, CA, USA) and examined using an FV1000 confocal laser-scanning microscope (Olympus, Tokyo, Japan).

### 2.12. Statistical Analysis

Data are presented as the mean ± standard deviation (SD). Statistical significance was evaluated by Student’s *t*-test. Differences were considered statistically significant at *p* < 0.05. 

## 3. Results

### 3.1. Anti-Viral Compound Library Screening

To identify novel anti-HEV candidates, an anti-viral compound library by TargetMol comprising 800 compounds with anti-virus bioactivity was screened using three HEV reporter systems: HEV-nanoKAZ, the HEV-GLuc replicon, and HEV-HiBiT (Figure 1). Ribavirin was used as a reference drug. In the primary screening all compounds were tested at 10 μM in parallel with a cell viability assay. PLC/PRF/5 cells were either inoculated with HEV-nanoKAZ in the presence of compounds or transfected with pHEV-GLuc replicon RNA or pHEV-HiBiT RNA followed by compound treatment (Figure 2). Luciferase activity was measured at 4 days post-inoculation or post-transfection (Figure 2). Compounds that met the predefined selection criteria (inhibition activity >80% and cell viability >80%) were advanced to secondary screening at 1 μM (Table 1). Compounds exhibiting >70% inhibition and >80% cell viability were defined as hits (Table 1).

**Figure 3:**
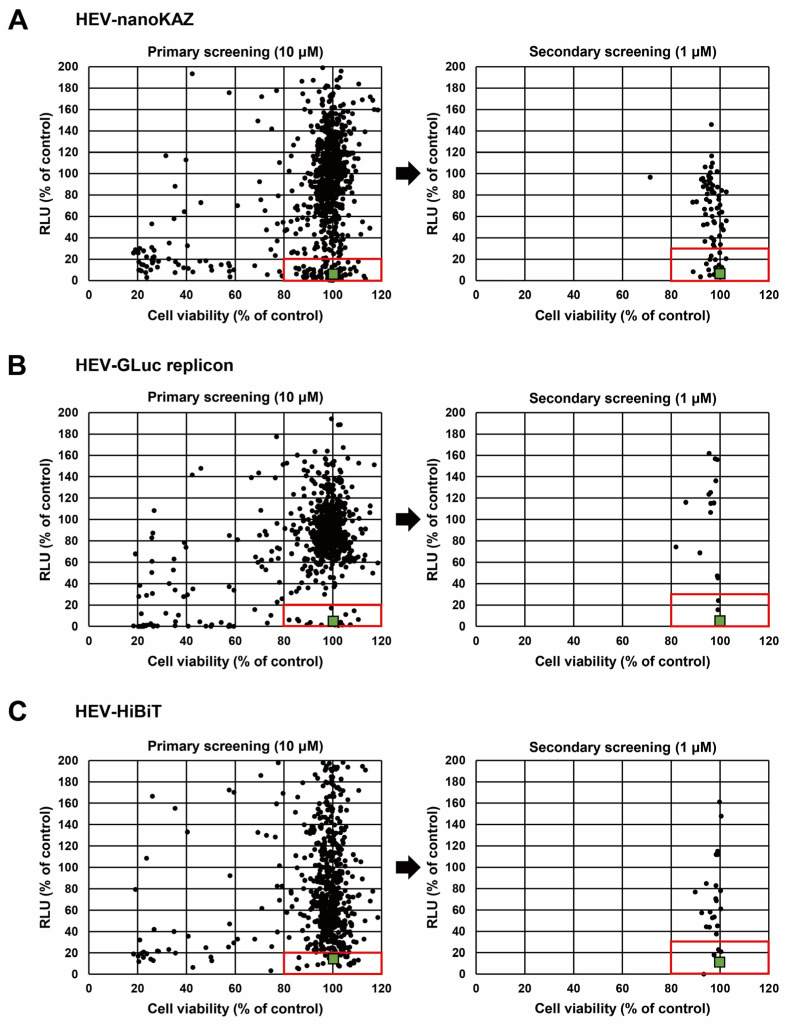
Screening of an anti-viral compound library (800 compounds using HEV-nanoKAZ (**A**), HEV-GLuc replicon (**B**), and HEV-HiBiT (**C**) assays. Primary screening was performed at 10 μM, with selection criteria of >80% inhibition and >80% cell viability (left panels). Compounds meeting these criteria were subjected to secondary screening at 1 μM, with selection criteria of >70% inhibition and >80% cell viability (right panels). Compounds that passed each screening step are indicated by red boxes. Ribavirin (green square) served as a reference drug. Values are normalized to untreated controls and presented as mean ± standard deviation (SD) of duplicate wells. RLU, relative light units.

Seventeen compounds were identified in the HEV-nanoKAZ assay (Figure 3, upper panel), two in the HEV-GLuc replicon assay (Figure 3, middle panel), and four in the HEV-HiBiT assay (Figure 3, lower panel). In the context of chronic HEV infection, current European Association for the Study of the Liver (EASL) and Japanese guidelines recommend at least 12 weeks of ribavirin therapy [24,26]. Given this prolonged treatment duration, compounds with known anticancer activity were excluded from further evaluation due to potential safety concerns. Ritonavir was also identified as a hit in the HEV-nanoKAZ assay; however, given its prior identification in our Food and Drug Administration (FDA)-approved drug library screening [30] and being shown to exert an additive effect with ribavirin in clearing HEV in cell culture [37] it was excluded from further analysis in this study. Finally, the selected compounds for further evaluation included four from the HEV-nanoKAZ assay (elbasvir, cenicriviroc, samatasvir, and hydroxychloroquine sulfate), two from HEV-GLuc replicon assay (azvudine and bemnifosbuvir), and four from the HEV-HiBiT assay (azvudine, bemnifosbuvir hemisulfate, bemnifosbuvir, and RO8191) (Table 1).

### 3.2. Anti-HEV Activity of Hit Compounds

To validate the inhibitory activity of the selected hit compounds, dose–response analyses were performed over a concentration range of 0.1–20 μM. Luciferase activity was measured at 4 days post-inoculation or post-transfection, with cells treated with 1% DMSO serving as controls.

**Figure 4:**
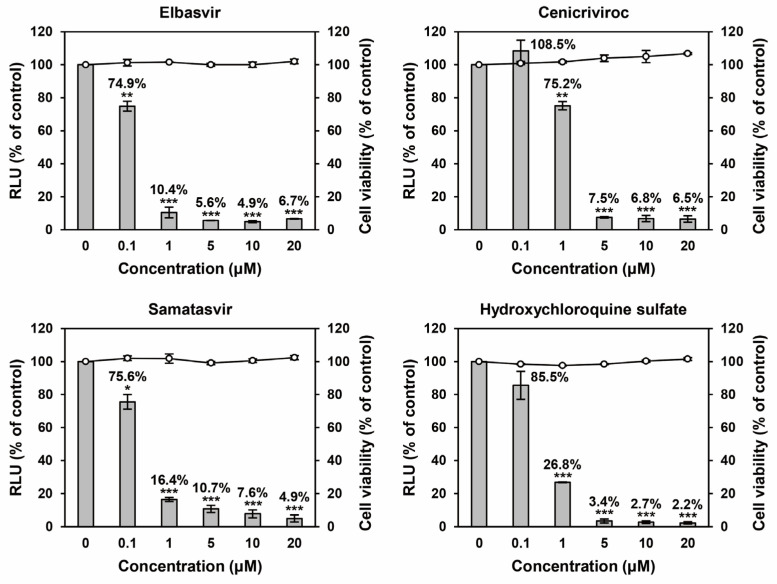
Validation of anti-HEV activity of selected hit compounds using the HEV-nanoKAZ assay. The inhibitory effects of elbasvir, cenicriviroc, samatasvir, and hydroxychloroquine sulfate against HEV were evaluated at the indicated concentrations (bar graphs, left axis), with corresponding cell viability measured in parallel (line graphs, right axis). Values are normalized to untreated controls. Data are presented as mean ± SD of duplicate wells. * *p* < 0.05; ** *p* < 0.01; *** *p* < 0.001.

In the HEV-nanoKAZ assay, elbasvir reduced luciferase activity to 74.9%, 10.4%, 5.6%, 4.9%, and 6.7% at 0.1, 1, 5, 10, and 20 μM, respectively (*p* < 0.01 or *p* < 0.001). Cenicriviroc showed no inhibitory effect at 0.1 μM (108.5%) but reduced luciferase activity to 75.2%, 7.5%, 6.8%, and 6.5% at 1, 5, 10, and 20 μM, respectively (*p* < 0.01 or *p* < 0.001). Samatasvir exhibited dose-dependent inhibition, decreasing luciferase activity to 75.6%, 16.4%, 10.7%, 7.6%, and 4.9% at 0.1, 1, 5, 10, and 20 μM, respectively (*p* < 0.05 or *p* < 0.001). Similarly, hydroxychloroquine sulfate decreased luciferase activity to 85.5%, 26.8%, 3.4%, 2.7%, and 2.2% across the same concentration range (*p* < 0.001) (Figure 4). No cytotoxicity was observed for any compound at concentrations up to 20 μM, with cell viability maintained above 80% (Figure 4).

**Figure 5:**
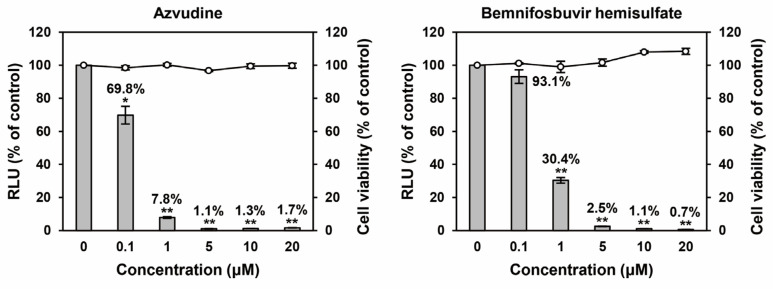
Validation of anti-HEV activity of selected hit compounds using the HEV-GLuc assay. The inhibitory effects of azvudine and bemnifosbuvir hemisulfate against HEV were evaluated at the indicated concentrations (bar graphs, left axis), with corresponding cell viability measured in parallel (line graphs, right axis). Values are normalized to untreated controls. Data are presented as mean ± SD of duplicate wells. * *p* < 0.05; ** *p* < 0.001.

In the HEV-GLuc replicon assay, azvudine reduced luciferase activity to 69.8%, 7.8%, 1.1%, 1.3%, and 1.7% at 0.1, 1, 5, 10, and 20 μM, respectively (*p* < 0.05 or *p* < 0.001). Bemnifosbuvir hemisulfate exhibited dose-dependent inhibition, decreasing luciferase activity to 93.1%, 30.4%, 2.5%, 1.1%, and 0.7% across the same concentration range (*p* < 0.001) (Figure 5). No cytotoxicity was observed at concentrations up to 20 μM (Figure 5).

**Figure 6:**
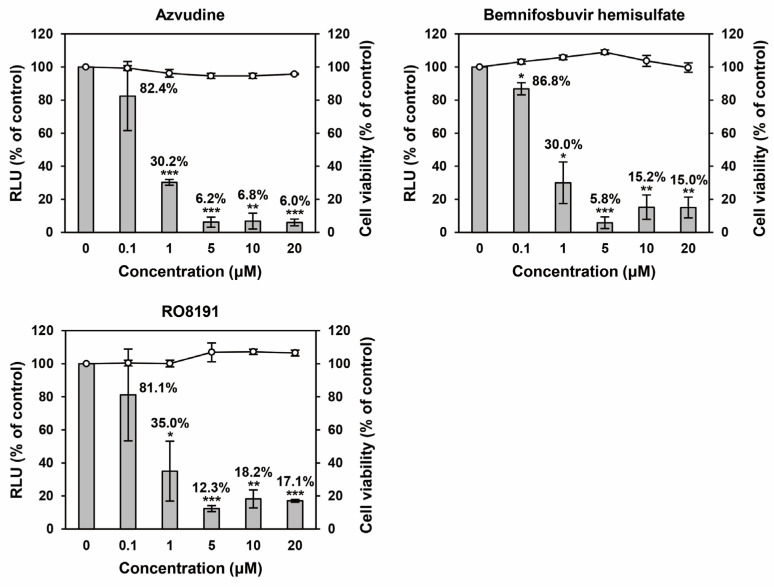
Validation of anti-HEV activity of selected hit compounds using the HEV-HiBiT assay. The inhibitory effects of azvudine, bemnifosbuvir hemisulfate, and RO8191 against HEV were evaluated at the indicated concentrations (bar graphs, left axis), with corresponding cell viability measured in parallel (line graphs, right axis). Values are normalized to untreated controls. Data are presented as mean ± SD of duplicate wells. * *p* < 0.05; ** *p* < 0.01; *** *p* < 0.001.

In the HEV-HiBiT assay, bemnifosbuvir hemisulfate and bemnifosbuvir represent closely related forms of the same compound; therefore, bemnifosbuvir hemisulfate was selected for further evaluation. Azvudine reduced luciferase activity to 82.4%, 30.2%, 6.2%, 6.8%, and 6.0% at 0.1, 1, 5, 10, and 20 μM, respectively (*p* < 0.01 or *p* < 0.001). Bemnifosbuvir hemisulfate decreased luciferase activity to 86.8%, 30.0%, 5.8%, 15.2%, and 15.0% across the same concentration range (*p* < 0.05, *p* < 0.01, or *p* < 0.001). RO8191 decreased luciferase activity to 81.1%, 35.0%, 12.3%, 18.2%, and 17.1% at 0.1, 1, 5, 10, and 20 μM, respectively (*p* < 0.05, *p* < 0.01, or *p* < 0.001) (Figure 6). No cytotoxicity was observed for any compound at concentrations up to 20 μM, with cell viability remaining above 80% (Figure 6).

### 3.3. Evaluation of Antiviral Efficacy of Selected Hit Compounds in an HEV Cell Culture System (Acute Infection Model)

**Figure 7:**
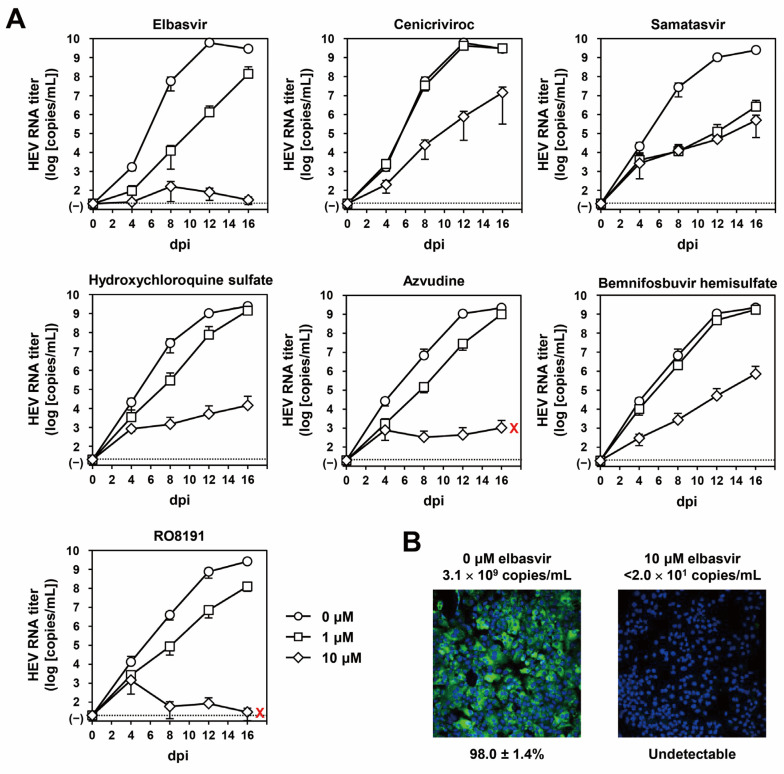
Evaluation of the antiviral efficacy of selected hit compounds against HEV using an in vitro acute infection model. (**A**) HEV growth kinetics were monitored for 16 days in PLC/PRF/5 cells inoculated with genotype 3 HEV (HEV-3) in the presence of each compound at 1 or 10 μM. Red crosses represent cytotoxicity found during cell culture. Data are presented as mean ± SD of quadruplicate wells. The dotted horizontal line represents the limit of detection by the real-time RT-PCR assay used in this study (2 × 10^1^ RNA copies/mL). dpi, days post-inoculation. (**B**) Immunofluorescence staining of the 10 μM elbasvir-treated cells from day 16, compared with untreated controls, using an anti-ORF2 monoclonal antibody (MAb). ORF2, green; DAPI, blue. Data are presented as mean ± SD. HEV-infected cells were quantified from two visual fields, with at least 200 cells counted per field. Results from one of the two representative wells are shown.

To evaluate the antiviral efficacy of the selected hit compounds in an acute infection model, virus growth kinetics were assessed in PLC/PRF/5 cells inoculated with wild-type HEV-3 in the presence of 1 or 10 μM of each compound. HEV RNA titers in culture supernatants were monitored for 16 days. In untreated controls for all compounds, viral RNA levels increased over time, reaching ~10^8^ copies/mL by 16 days post-inoculation (dpi) (Figure 7A).

Elbasvir exhibited a clear dose-dependent inhibitory effect on viral replication. At 1 μM, elbasvir had minimal impact, with HEV RNA titers increasing to 1.4 × 10^8^ copies/mL by 16 dpi. In contrast, treatment with 10 μM elbasvir reduced HEV RNA titer in culture supernatants to undetectable level by 16 dpi (Figure 7A). On the other hand, cenicriviroc, samatasvir, hydroxychloroquine sulfate, azvudine, bemnifosbuvir hemisulfate showed minimal or no inhibitory effects at 1 μM, with HEV RNA titer increasing over time to levels comparable to untreated controls. However, increasing the concentration to 10 μM resulted in only modest improvements in antiviral efficacy. Notably, azvudine and RO8191 exhibited cytotoxic effects at this concentration (Figure 7A).

At 16 dpi, cells treated with 10 μM elbasvir and untreated control cells were subjected to immunofluorescence analysis to assess intracellular HEV ORF2 expression. Nearly all untreated control cells were positive for ORF2 (98.0 ± 1.4%), whereas ORF2 expression was undetected in cells treated with 10 μM elbasvir (Figure 7B), consistent with the observed suppression of viral replication.

Taken together, these results identify elbasvir as the only compound exhibiting robust antiviral activity in this acute in vitro HEV infection model.

### 3.4. Elbasvir Suppresses HEV Growth in a Chronic Infection Co-Culture Model

To mimic conditions of chronic HEV infection, a co-culture system was established by mixing naïve PLC/PRF/5 cells with chronically infected PLC/PRF/5 cells that continuously produce high titers of virus (>10^8^ copies/mL). This system was used to further evaluate the efficacy of elbasvir. At the initiation of treatment, HEV RNA titers in culture supernatants were >10^4^ copies/mL. Virus growth kinetics were monitored for 16 days following treatment with various concentrations of elbasvir.

**Figure 8:**
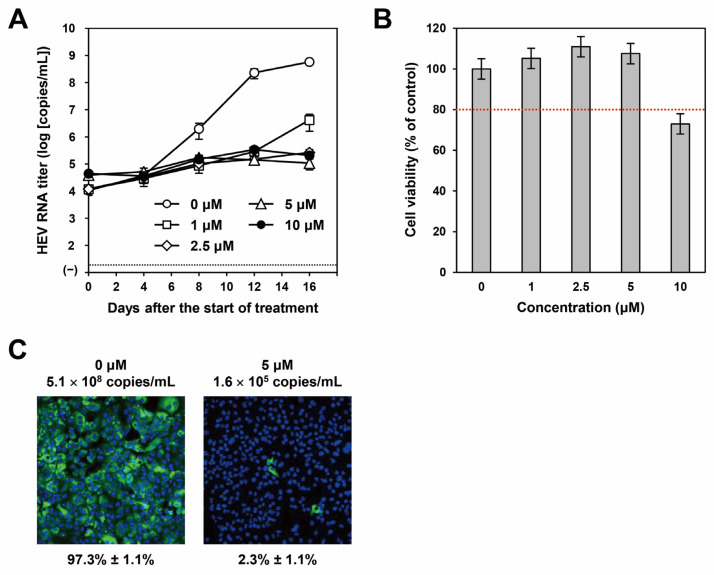
Evaluation of the inhibitory effect of elbasvir on HEV growth in an in vitro chronic infection model. (**A**) HEV growth kinetics were observed for 16 days in PLC/PRF/5 cells robustly producing HEV in the presence of varying concentrations of elbasvir. Data are presented as mean ± SD of quadruplicate wells. The dotted horizontal line represents the limit of detection of the real-time RT-PCR assay used in this study (2 × 10^1^ RNA copies/mL). (**B**) Post-culture cell viability was assessed at day 16 using treated cells in comparison with untreated controls to exclude cytotoxic effects. The viability threshold (80%) is represented by a dotted red horizontal line. Data are presented as mean ± SD of duplicate wells. (**C**) Immunofluorescence staining of the 5 μM elbasvir-treated cells at day 16, compared with untreated controls, using an anti-ORF2 MAb. ORF2, green; DAPI, blue. Data are presented as mean ± SD. HEV-infected cells were quantified from two visual fields, with at least 200 cells counted per field. Results from one of the two representative wells are shown.

In untreated control cells, HEV RNA titers in culture supernatants increased over time and reached a plateau (~10^8^ copies/mL) by day 12 after treatment initiation (Figure 8A). At 1 μM, elbasvir did not effectively inhibit HEV growth, with HEV RNA titers gradually increasing to 4.2 × 10^6^ copies/mL by day 16. In contrast, treatment with 2.5, 5, or 10 μM elbasvir suppressed virus growth, maintaining HEV RNA titers near baseline throughout the observation period (Figure 8A).

To assess potential cytotoxicity, post-culture cell viability was measured at day 16. Viability for 2.5 and 5 μM-treated cells was comparable to controls (110.9% and 107.5%, respectively), whereas 10 μM elbasvir reduced cell viability to 73.0% (Figure 8B), indicating cytotoxicity under prolonged exposure.

At day 16, the cells treated with 5 μM elbasvir were subjected to immunofluorescence analysis to assess intracellular ORF2 expression. ORF2 expression was detected in nearly all untreated control cells (97.3 ± 1.1%; Figure 8C, left panel), but was markedly reduced in cells treated with 5 μM elbasvir (2.3% ± 1.1%; Figure 8C, right panel), consistent with the viral growth kinetics (Figure 8A).

Together, these results demonstrate that elbasvir exerts potent and sustained antiviral activity against HEV in both acute and chronic in vitro infection models.

### 3.5. Elbasvir in Combination with Ribavirin Achieves Sustained Suppression of HEV in a Chronic Infection Co-Culture Model

**Figure 9:**
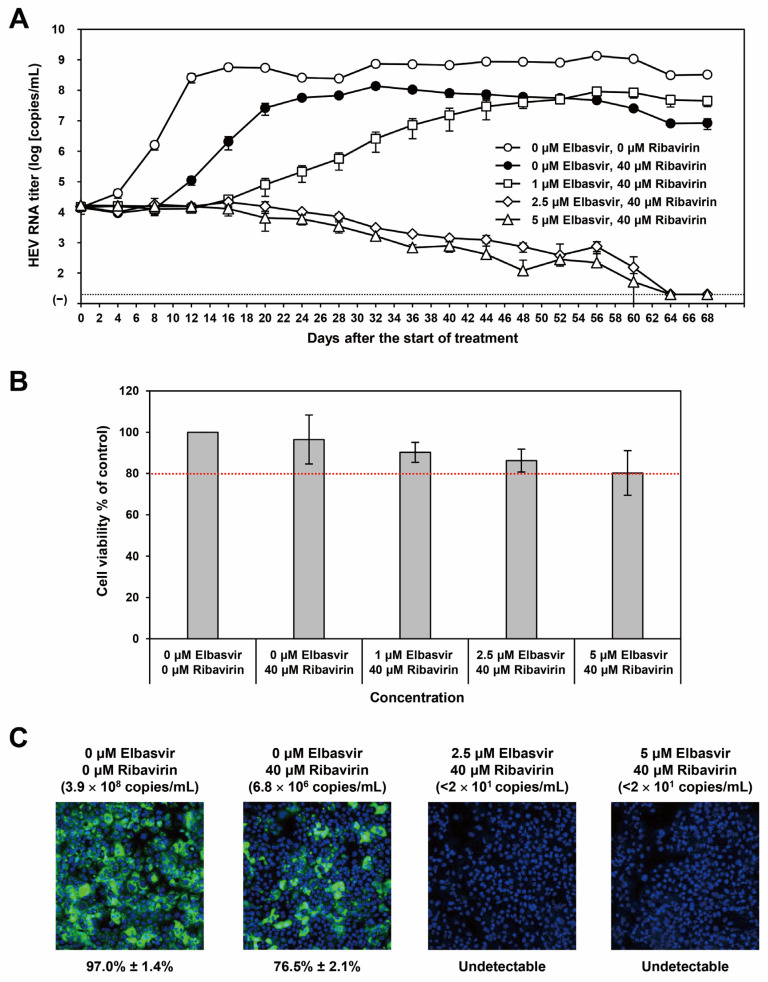
Evaluation of the combined antiviral effect of elbasvir and ribavirin in an in vitro chronic infection model. (**A**) HEV growth kinetics were monitored for 68 days in PLC/PRF/5 cells robustly producing HEV in the presence of elbasvir at various concentrations combined with ribavirin (40 μM), compared with ribavirin monotherapy (40 μM). Data are presented as mean ± SD of quadruplicate wells. The dotted horizontal black line represents the limit of detection by the real-time RT-PCR assay used in this study (2 × 10^1^ RNA copies/mL). (**B**) Post-culture cell viability was assessed at day 68 using drug-treated cells in comparison with untreated controls to confirm that the observed antiviral effects were not due to cytotoxicity. The viability threshold (80%) is represented by a dotted red horizontal line. Data are presented as mean ± SD of triplicate wells. (**C**) Immunofluorescence staining of the drug-treated cells collected at day 68 in comparison with untreated controls, using an anti-ORF2 MAb. ORF2, green; DAPI, blue. HEV-infected cells were quantified from two visual fields, with at least 200 cells counted per field. Results from one of the two representative wells are shown.

To enhance the antiviral efficacy of elbasvir, its combination with ribavirin was evaluated using the chronic infection co-culture model. Elbasvir concentrations (1, 2.5, and 5 μM) were selected based on single-treatment efficacy in chronic infection co-culture model (Figure 8A) and the post-culture cell viability assay (Figure 8B), while ribavirin was used at 40 μM according to our previous reports [30,37]. At the initiation of treatment, HEV RNA titers in culture supernatants exceeded 10^4^ copies/mL. Viral growth kinetics were monitored for 68 days following treatment with the elbasvir–ribavirin combination and compared with ribavirin monotherapy (Figure 9A).

In untreated control cultures, HEV RNA levels increased over time and reached a plateau (>10^8^ copies/mL) from day 12 onward. Ribavirin monotherapy transiently suppressed virus growth during the first week; however, viral RNA levels subsequently rebounded, approaching those of untreated controls. In contrast, combination treatment enhanced the antiviral effect of elbasvir.

At day 16, treatment with 1 μM elbasvir alone yielded 4.2 × 10^6^ copies/mL (Figure 8A), whereas combination with ribavirin reduced HEV RNA titers to 2.6 × 10^4^ copies/mL (Figure 9A). Similarly, 2.5 μM elbasvir alone resulted in 2.7 × 10^5^ copies/mL (Figure 8A), compared with 2.1 × 10^4^ copies/mL in combination with ribavirin (Figure 9A). At 5 μM, elbasvir alone yielded 1.1 × 10^5^ copies/mL, whereas combination treatment further reduced HEV RNA titers in culture supernatants to 1.3 × 10^4^ copies/mL (Figure 9A).

Over prolonged observation, the combination of 1 μM elbasvir with ribavirin was insufficient to sustain viral suppression, with RNA titers increasing from day 20 and reaching 4.5 × 10^7^ copies/mL by day 68. In contrast, combination of 2.5 or 5 μM elbasvir with ribavirin achieved sustained inhibition, with HEV RNA levels gradually declining to below the limit of detection from day 64 onward (Figure 9A). Cell viability at day 68 remained above the 80% threshold for all combination treatments (90.2%, 86.2%, and 80.2% for combination of ribavirin with 1, 2.5, and 5 μM elbasvir, respectively; Figure 9B), indicating that the observed antiviral effects were not attributable to cytotoxicity.

Immunofluorescence analysis performed at day 68 demonstrated abundant HEV ORF2 expression in untreated control cells (97.0% ± 1.4%) and partial reduction following ribavirin monotherapy (76.5% ± 2.1%). In contrast, ORF2 expression was undetectable in cells treated with the combination of 40 μM ribavirin and either 2.5 or 5 μM elbasvir (Figure 9C), consistent with the viral growth kinetics (Figure 9A).

Collectively, these results demonstrate that combination treatment with elbasvir and ribavirin enables sustained suppression and eventual clearance of HEV in a chronic in vitro infection model.

### 3.6. Elbasvir and Ribavirin Combination Exhibits Additive Antiviral Effects

**Figure 10:**
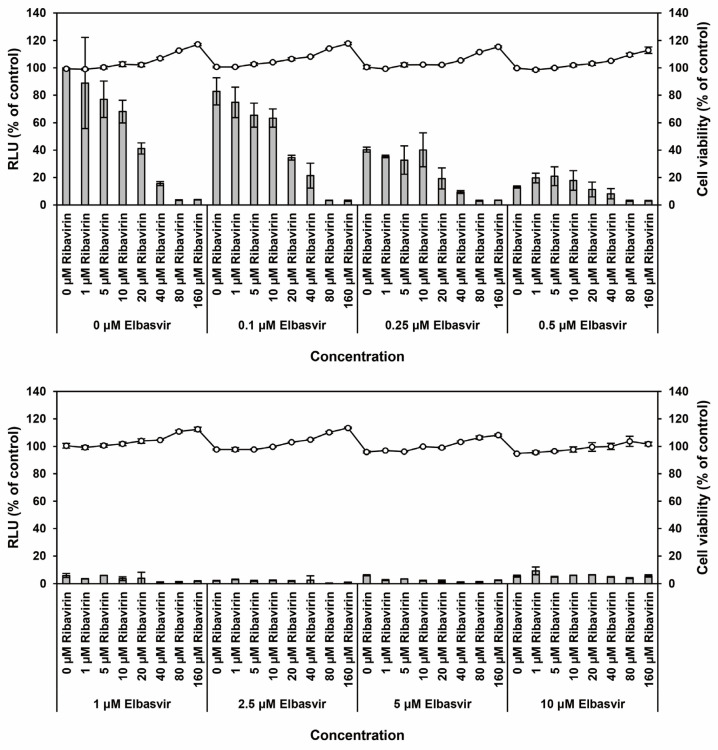
Validation of the anti-HEV activity of the elbasvir and ribavirin combination. The effects of combination treatment were evaluated using the HEV-nanoKAZ assay (bar graphs, left axis), with corresponding cell viability measured in parallel (line graphs, right axis). PLC/PRF/5 cells were treated with the indicated concentrations of elbasvir and ribavirin combination. Luciferase activity and cell viability are expressed as percentages relative to the untreated controls. For clarity, the full set of 64 drug combinations (0–10 μM elbasvir and 0–160 μM ribavirin) is divided into two panels (upper and lower). Data are presented as mean ± SD of duplicate wells.

To evaluate the interaction between elbasvir and ribavirin, drug combination analysis was performed using the browser-independent web application SynergyFinder (version 3.0) [36]. Antiviral responses were assessed across 64 drug combinations using the HEV-nanoKAZ assay. The concentrations for elbasvir were 0, 0.1, 0.25, 0.5, 1, 2.5, 5, and 10 μM, while those for ribavirin were 0, 1, 5, 10, 20, 40, 80, 160 μM. The graph was divided into two parts for clarity (Figure 10 upper and lower panels). The combinations decreased luciferase activity in a dose-dependent manner (Figure 10, bar graphs, left axis) without exerting any significant effects on the cellular proliferation or survival, as confirmed by cell viability assay (Figure 10, line graphs, right axis).

**Figure 11:**
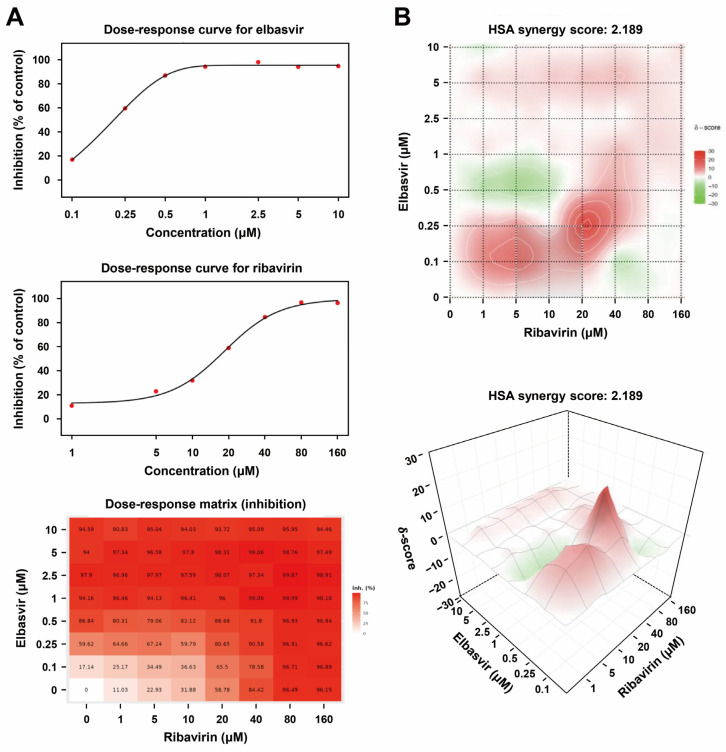
Quantification and visualization of the synergy of elbasvir and ribavirin combination using SynergyFinder (version 3.0) [36]. (**A**) Dose–response curves for elbasvir (0–10 μM; upper left panel) and ribavirin (0–160 μM; middle left panel), and dose–response matrix for the 64 drug combinations (lower left panel), calculated based on inhibition of intracellular luciferase activity in HEV-nanoKAZ-inoculated PLC/PRF/5 cells treated with the indicated drug concentrations. (**B**) Synergy maps generated using the highest single agent (HSA) model, visualized in two-dimensional (upper panel) and three-dimensional (lower panel) formats. Synergy scores indicate drug interactions, where scores (<−10 indicate antagonism, −10 to 10 indicate additive effects, and >10 indicate synergy).

To calculate the degree of synergism of the combination, the inhibition responses from a total of 64 combinations tested in Figure 10 were applied to SynergyFinder (version 3.0). Dose–response plots of phenotypic responses for the single drug (i.e., response measurements, when the other drug concentrations were 0), fitted by four-parameter logistic curve, are presented for elbasvir (Figure 11A, upper left panel) and ribavirin (Figure 11A, middle left panel). The observed drug combination responses (Figure 11A, lower left panel) were compared with the expected combination responses calculated by the highest single agent (HSA) reference model to determine the degree of synergism of elbasvir and ribavirin combination treatment, yielding a synergy score of 2.189, indicating an additive interaction. Results were visualized as two- (Figure 11B, upper right panel) and three-dimensional (Figure 11B, lower right panel) synergy maps.

These findings indicate that elbasvir and ribavirin exert additive antiviral effects without cytotoxicity.

### 3.7. Elbasvir Inhibits HEV Internalization

**Figure 12:**
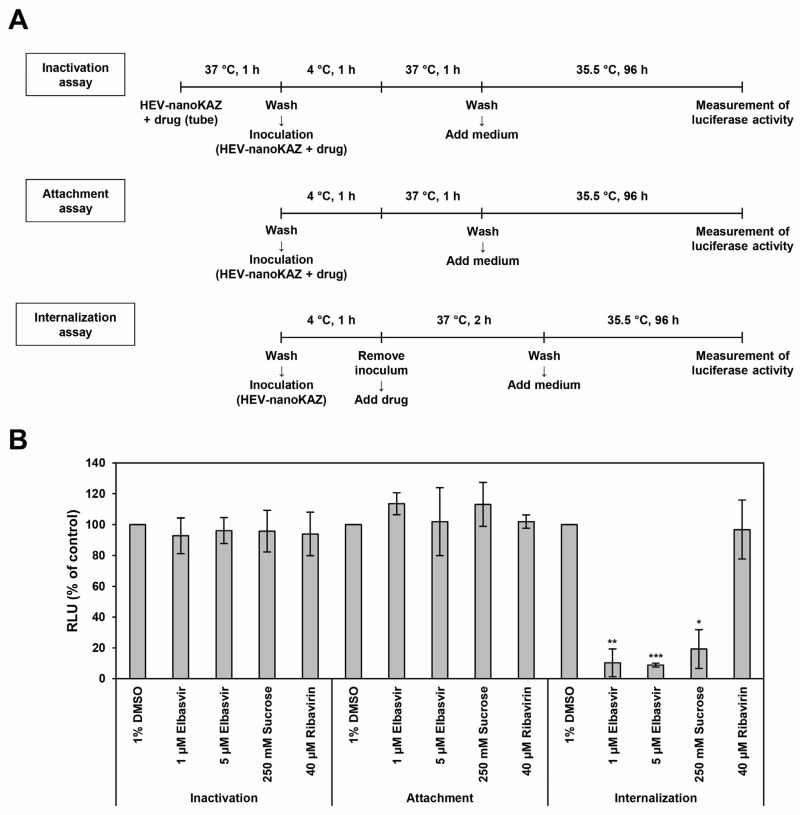
Identification of the HEV life cycle step targeted by elbasvir using a time-of-addition assay. (**A**) Schematic overview of the assay design, including viral inactivation, attachment, and internalization steps, performed using HEV-nanoKAZ. (**B**) PLC/PRF/5 cells were inoculated with HEV-nanoKAZ, and elbasvir (1 or 5 μM) was added at the indicated time points corresponding to inactivation, attachment, or internalization phases. Luciferase activity was measured and normalized to untreated controls. Sucrose (250 mM) and ribavirin (40 μM) were included as reference treatments. Data are present as mean ± SD of two independent experiments. * *p* < 0.05; ** *p* < 0.01; *** *p* < 0.001.

Elbasvir selectively reduced luciferase activity in the HEV-nanoKAZ assay, suggesting an effect on an early step in the HEV life cycle. To identify the targeted stage, a time-of-addition assay was performed under viral inactivation, attachment, and internalization conditions (Figure 12A). Sucrose, an inhibitor of clathrin-mediated endocytosis, and ribavirin, an inhibitor of HEV RNA replication, were included as reference compounds.

Preincubation of elbasvir (1 or 5 μM) with HEV-nanoKAZ prior to inoculation did not reduce luciferase activity in the viral inactivation assay (92.8% and 96.2%, respectively) (Figure 12B), indicating that elbasvir did not inactivate HEV in a cell-free state to prevent subsequent infection. Similarly, elbasvir had no effect in the viral attachment assay (113.5% and 102.0%, respectively) (Figure 12B). In contrast, elbasvir significantly reduced luciferase activity in the internalization assay at both concentrations (10.3%, *p* < 0.01; 8.8%, *p* < 0.001), comparable to sucrose treatment (19.2%, *p* < 0.05) (Figure 12B). Ribavirin did not reduce luciferase activity under any condition tested (Figure 12B). These results indicate that elbasvir inhibits HEV internalization.

## 4. Discussion

Although acute hepatitis E is typically self-limiting in immunocompetent individuals, immunocompromised patients often fail to clear the infection, leading to chronic hepatitis E. Antiviral therapy is therefore required for these patients, as well as for those with acute severe or fulminant disease [24,25]. Ribavirin, which is currently used off-label for the treatment of HEV infection, is associated with clinically significant adverse effects, including anemia [26,27,28], highlighting the need for safer and more effective therapeutic options.

One promising strategy for the development of novel anti-viral agents is to target distinct steps of the viral life cycle to enhance efficacy and reduce the risk of drug resistance [38], including for the treatment of HEV infection. Accordingly, we screened an anti-viral compound library comprising 800 compounds using our recently established stage-specific HEV reporter systems (Figure 1 and Figure 2), enabling systematic identification of candidate compounds acting at different stages of the HEV life cycle. In the context of chronic HEV infection, current EASL and Japanese guidelines recommend at least 12 weeks of ribavirin therapy [24,25]. Given this prolonged treatment duration, compounds with known anticancer activity were excluded from further evaluation due to potential safety concerns.

The final set of selected hits included four compounds from the HEV-nanoKAZ assay (elbasvir, cenicriviroc, samatasvir, and hydroxychloroquine sulfate), two from the HEV-GLuc replicon assay (azvudine and bemnifosbuvir hemisulfate), and four from the HEV-HiBiT assay (azvudine, bemnifosbuvir hemisulfate, bemnifosbuvir, and RO8191) (Figure 3, Table 1). Given that bemnifosbuvir hemisulfate and bemnifosbuvir represent closely related forms of the same compound, only bemnifosbuvir hemisulfate was selected for further evaluation.

Prior to evaluation in cell culture models, the antiviral activity of the selected hit compounds was validated using the respective HEV reporter systems (HEV-nanoKAZ, Figure 4; HEV-GLuc replicon, Figure 5; and HEV-HiBiT, Figure 6), with cell viability assessed in parallel. All compounds exhibited dose-dependent inhibition of luciferase activity without significant cytotoxicity, indicating that the observed anti-HEV activity was not due to cytotoxicity. Based on these results, appropriate concentrations were selected for subsequent cell culture experiments.

Each compound was then evaluated at 1 and 10 μM in PLC/PRF/5 cells inoculated with wild-type HEV-3, and virus growth kinetics were monitored for 16 days. While treatment at 1 μM was insufficient to suppress HEV growth over prolonged culture, increasing the concentration to 10 μM enhanced antiviral efficacy across compounds, ranging from modest effects for cenicriviroc, samatasvir, hydroxychloroquine sulfate, and bemnifosbuvir hemisulfate, to potent inhibition by elbasvir. In contrast, azvudine and RO8191 exhibited cytotoxicity at 10 μM (Figure 7A). The robust anti-HEV activity of elbasvir was further corroborated by the complete absence of intracellular HEV ORF2 expression in immunofluorescence analysis (Figure 7B).

Notably, although several compounds exhibited strong inhibitory effects in short-term reporter assays, most failed to sustain antiviral activity during prolonged culture using wild-type virus. Elbasvir was the only compound that maintained durable suppression under these conditions, highlighting its distinct advantage over other candidates identified through initial screening. Furthermore, while low concentration (1 μM) was sufficient to demonstrate potent inhibition in the reporter assay (HEV-nanoKAZ), a higher concentration (10 μM) was required to achieve effective suppression in cell culture. These findings underscore the importance of validating candidate compounds in long-term infection models using wild-type virus, as short-term reporter assays alone may overestimate antiviral efficacy. This approach is particularly relevant in the context of HEV infection, for which prolonged treatment is recommended, highlighting the need to assess sustained antiviral activity when evaluating potential therapeutic candidates.

To further assess its anti-HEV potential in a clinically relevant context, elbasvir was evaluated in a chronic infection model, given that antiviral therapy is primarily indicated for patients with chronic HEV infection. A co-culture system was employed in which naïve PLC/PRF/5 cells were mixed with chronically infected cells that continuously produce high titers of virus. Treatment with elbasvir at varying concentrations was initiated when HEV RNA levels in culture supernatants exceeded 10^4^ copies/mL, and viral growth kinetics were monitored for 16 days. Consistent with the acute infection model (Figure 8A), 1 μM elbasvir was insufficient to suppress virus growth. In contrast, 2.5, 5, and 10 μM elbasvir achieved comparable suppression, maintaining HEV RNA levels near baseline throughout the observation period (Figure 8A). However, post-culture cell viability analysis indicated that the apparent antiviral effect at 10 μM was likely influenced by cytotoxicity (Figure 8B), emphasizing the importance of incorporating post-treatment viability assessment in long-term antiviral studies to accurately distinguish antiviral effect from cytotoxicity, thus avoiding overestimation of antiviral efficacy. The antiviral effect of elbasvir at non-cytotoxic concentration was supported by immunofluorescence analysis, which showed a marked reduction in intracellular HEV ORF2 expression in cells treated with 5 μM elbasvir compared with untreated control cells at 16 days after the treatment initiated (Figure 8C).

To further enhance antiviral efficacy, elbasvir was combined with ribavirin in the same chronic infection model. Viral growth kinetics were monitored for 68 days. Combination treatment consistently improved antiviral activity. At 16 days after treatment initiation, HEV RNA levels were 10- to 200-folds lower in the elbasvir–ribavirin combination groups compared with the corresponding elbasvir monotherapy groups (Figure 9A vs. Figure 8A) or ribavirin monotherapy (Figure 9A). At this time point, 1 μM elbasvir alone yielded viral RNA levels comparable to those observed with ribavirin alone, whereas 2.5 and 5 μM elbasvir demonstrated stronger inhibition (Figure 9A), consistent with immunofluorescence findings (Figure 9C). Importantly, these enhanced antiviral effects were achieved without significant cytotoxicity, as confirmed by post-culture cell viability assays (Figure 9B). In addition, evaluation of 64 elbasvir–ribavirin combinations across a wide concentration range (0–10 μM elbasvir and 0–160 μM ribavirin) confirmed minimal impact on cell viability (Figure 10). Of note, while the short-term reporter assay suggested minimal cytotoxicity of the elbasvir–ribavirin combinations (Figure 10), prolonged exposure in the chronic infection model revealed cytotoxicity effects at 10 μM elbasvir (Figure 8B). Given that treatment of chronic HEV infection generally requires extended drug administration, long-term culture experiments using wild-type HEV are important not only for evaluating sustained antiviral efficacy but also for accurately assessing cumulative cytotoxicity. Together, these findings support the efficacy and safety of the elbasvir–ribavirin combination approach.

Mechanistically, elbasvir selectively reduced luciferase activity in the HEV-nanoKAZ assay, indicating an effect on an early stage of the viral life cycle. Time-of-addition analysis further demonstrated that elbasvir specifically inhibits viral internalization, with no effect on viral inactivation or attachment (Figure 12B). In contrast, ribavirin inhibits HEV RNA replication, indicating that the two agents target distinct steps in the viral life cycle. Targeting complementary steps of infection likely underlies the enhanced efficacy observed with combination treatment. This approach may also reduce the likelihood of resistance, as viral escape would require simultaneous adaptation to multiple mechanisms, and may permit dose reduction, thereby minimizing toxicity. Consistent with this interpretation, synergy analysis yielded an additive interaction (HSA score = 2.189, Figure 11B), indicating the absence of antagonism between the two drugs. Together, these findings support the potential of elbasvir–ribavirin combination therapy as a rational and effective strategy for HEV treatment.

A suitable positive control for the internalization assay was included using sucrose, a well-established inhibitor of clathrin-mediated endocytosis. In contrast, the availability of well-characterized positive controls for HEV viral inactivation and attachment assays remains limited. Neutralizing antibodies against the HEV capsid protein, such as H6225, have previously been used in HEV neutralization assays [35,39,40,41,42]. However, these antibodies primarily target non-enveloped HEV (neHEV), whereas the present study mainly utilized quasi-enveloped HEV (eHEV) in the cell culture system, limiting their applicability in our experimental setting. In addition, although several host factors have been proposed to contribute to HEV attachment [43], the definitive cellular receptor(s) for HEV have not yet been fully established, and no widely accepted specific attachment inhibitor is currently available. Therefore, the limited availability and applicability of appropriate controls for viral inactivation and attachment represent a limitation of the current HEV experimental system.

Validation of the antiviral activity of elbasvir using primary human hepatocytes would further strengthen the translational relevance of this study. However, because the present study was designed not only for initial antiviral screening but also for long-term evaluation of sustained antiviral efficacy and cytotoxicity, we employed the PLC/PRF/5 hepatoma cell line, which supports robust and reproducible long-term HEV propagation and has been widely used for HEV antiviral and mechanistic studies. In contrast, primary human hepatocytes have limited availability, donor-to-donor variability, and restricted long-term culture stability [44,45], making prolonged antiviral evaluation technically challenging.

Elbasvir exhibits substantially greater antiviral potency against HCV than against HEV. As an HCV NS5A inhibitor, elbasvir suppresses HCV RNA replication at nanomolar concentrations [46,47], whereas inhibition of HEV in our long-term culture system required micromolar concentrations. This difference may reflect a distinct mechanism of action against HEV, as our data suggest that elbasvir inhibits an early step of the HEV life cycle, specifically viral internalization, rather than viral RNA replication.

Clinically, elbasvir was primarily developed and approved in combination with grazoprevir for the treatment of chronic HCV infection [48,49,50,51,52]. Although ribavirin has occasionally been added to elbasvir–grazoprevir regimens in certain difficult-to-treat HCV patient populations [53,54] reports evaluating elbasvir–ribavirin combination therapy in the absence of grazoprevir remain limited. One possible explanation is that highly effective ribavirin-free direct-acting antiviral (DAA) regimens with improved safety profiles [55] have largely eliminated the clinical need for elbasvir–ribavirin dual therapy in HCV treatment.

Emerging evidence suggests that certain anti-HCV drugs may also exert activity against HEV. For example, the HCV non-structural protein 3/4A (NS3/4A) protease inhibitor telaprevir has been reported to inhibit HEV infection in co-infection models [56]. Subsequent studies have proposed that such inhibitors may act on host-encoded proteases required for HEV infection, as HEV lacks viral proteins with known protease activity. In particular, cellular cysteine proteases have been implicated in the establishment of HEV infection but not in viral RNA replication [57].

Consistent with these observations, our screening results showed that elbasvir selectively inhibited luciferase activity in the HEV-nanoKAZ assay (Figure 3A), which reflects early stages of the viral life cycle, without affecting luciferase activity in the HEV-GLuc replicon (Figure 3B) or HEV-HiBiT assays (Figure 3C), which represent RNA replication and later stages, respectively. Importantly, time-of-addition analysis further demonstrated that elbasvir specifically inhibits viral internalization, with no detectable effect on viral inactivation or attachment (Figure 12B). Together, these findings support a model in which elbasvir interferes with host-dependent processes required for HEV entry, particularly at the internalization step. Notably, both eHEV and neHEV rely on clathrin-mediated endocytosis for cellular entry [58,59], although the mechanism governing intracellular trafficking and uncoating might differ between the two particle forms. Since cell culture-derived HEV is predominantly released as eHEV, the findings of the present study are most directly applicable to eHEV infection.

Although elbasvir is known to target NS5A in HCV, HEV does not encode an NS5A homolog, suggesting that its anti-HEV activity is mediated through a distinct, likely host-targeted mechanism. Elbasvir may act by modulating host factors involved in clathrin-mediated endocytosis or membrane-trafficking, which are critical for HEV entry [58,59]. This hypothesis is supported by the comparable inhibitory effect observed with sucrose, a known inhibitor of clathrin-mediated endocytosis, in the internalization assay (Figure 12B). Alternatively, elbasvir may affect host protease activity required for viral entry, consistent with previous report implicating cellular cysteine proteases in HEV infection [57]. Further studies will be required to define the precise host targets and molecular mechanisms underlying elbasvir-mediated inhibition of HEV entry.

## 5. Conclusions

We identified elbasvir as a promising anti-HEV candidate through comprehensive screening of an anti-viral compound library. Elbasvir demonstrated sustained antiviral activity in both acute and chronic infection models, and its combination with ribavirin further enhanced antiviral efficacy without significant toxicity. Mechanistically, elbasvir inhibits viral internalization, complementing the inhibitory effect of ribavirin on RNA replication and supporting a step-specific combination strategy. However, this study has several limitations. First, all experiments were performed in vitro, and the precise host targets and molecular mechanisms underlying elbasvir-mediated inhibition remain to be fully elucidated. In addition, the antiviral activity of elbasvir was evaluated primarily using the PLC/PRF/5 hepatoma cell line. Although this system enables robust and reproducible long-term HEV propagation and is widely used for mechanistic and antiviral studies, hepatoma-derived cell lines may not fully recapitulate the physiological characteristics of primary human hepatocytes, including hepatic differentiation status, innate immune responses, drug metabolism capacity, and toxicity profiles. While primary human hepatocytes may provide greater physiological relevance, their limited long-term culture stability makes prolonged antiviral evaluation technically challenging. Furthermore, the limited availability and applicability of appropriate positive controls for viral inactivation and attachment assays represent an additional limitation of the current HEV experimental system. Future studies should therefore validate these findings using in vivo and physiologically relevant liver models to confirm the translational relevance of the observed antiviral effects, and to better predict potential clinical efficacy, while further defining the underlying molecular mechanisms, and assess the safety, efficacy, and resistance potential of elbasvir–ribavirin combination therapy across diverse HEV genotypes.

## Figures and Tables

**Table 1:** Results of the screening of an anti-viral compound library using HEV-nanoKAZ, HEV-GLuc replicon, and HEV-HiBiT assays.

Reporter	Primary Screening (10 μM)	Secondary Screening (1 μM)	Total Hits	Final Selected Hits
HEV-nanoKAZ	800 compounds Inhibition >80% Cell viability >80%	74 compounds Inhibition >70% Cell viability >80%	17 compounds	4 compounds Elbasvir Cenicriviroc Samatasvir Hydroxychloroquine sulfate
HEV-GLuc replicon	800 compounds Inhibition >80% Cell viability >80%	16 compounds Inhibition >70% Cell viability >80%	2 compounds	2 compounds Azvudine Bemnifosbuvir hemisulfate
HEV-HiBiT	800 compounds Inhibition >80% Cell viability >80%	24 compounds Inhibition >70% Cell viability >80%	4 compounds	4 compounds Azvudine Bemnifosbuvir hemisulfate Bemnifosbuvir RO8191

## Data Availability

All data are presented in the manuscript.

## Abbreviations

The following abbreviations are used in this manuscript:

HEV	hepatitis E virus
eHEV	membrane-associated HEV particles
neHEV	membrane-unassociated HEV particles
HCV	hepatitis C virus
NS5A	non-structural protein 5A
RNA	ribonucleic acid
kb	kilobases
UTR	untranslated region
ORF	open reading frame
MetY	methyltransferase and Y domain
FABD-like	fatty-acid binding domain-like domain
HVR	hypervariable region
X	X or macro domain
Hel	helicase domain
RdRp	RNA-dependent RNA polymerase domain
FBS	fetal bovine serum
DMSO	dimethyl sulfoxide
RLU	relative light unit
WST-8	water-soluble tetrazolium salt
PBS	phosphate-buffered saline
RT-PCR	real-time reverse transcription–polymerase chain reaction
HSA	highest single agent
v/v	volume/volume
BSA	bovine serum albumin
MAb	monoclonal antibody
DAPI	4’,6-diamidino-2-phenylindole
SD	standard deviation
EASL	the European Association for the Study of the Liver
FDA	Food and Drug Administration
dpi	days post-inoculation
NS3/4A	non-structural protein 3/4A
DAA	Direct-acting antiviral
